# Controlling type I error rates in multi-arm clinical trials: A case for the false discovery rate

**DOI:** 10.1002/pst.2059

**Published:** 2020-08-12

**Authors:** James M. S. Wason, David S. Robertson

**Affiliations:** 1 Population Health Sciences Institute, Faculty of Medical Sciences, Newcastle University, Newcastle upon Tyne, UK; 2 MRC Biostatistics Unit, University of Cambridge, Cambridge, UK

**Keywords:** error rate, false discovery rate, family-wise error rate, multi-arm trials

## Abstract

Multi-arm trials are an efficient way of simultaneously testing several experimental treatments against a shared control group. As well as reducing the sample size required compared to running each trial separately, they have important administrative and logistical advantages. There has been debate over whether multi-arm trials should correct for the fact that multiple null hypotheses are tested within the same experiment. Previous opinions have ranged from no correction is required, to a stringent correction (controlling the probability of making at least one type I error) being needed, with regulators arguing the latter for confirmatory settings. In this article, we propose that controlling the false-discovery rate (FDR) is a suitable compromise, with an appealing interpretation in multi-arm clinical trials. We investigate the properties of the different correction methods in terms of the positive and negative predictive value (respectively how confident we are that a recommended treatment is effective and that a non-recommended treatment is ineffective). The number of arms and proportion of treatments that are truly effective is varied. Controlling the FDR provides good properties. It retains the high positive predictive value of FWER correction in situations where a low proportion of treatments is effective. It also has a good negative predictive value in situations where a high proportion of treatments is effective. In a multi-arm trial testing distinct treatment arms, we recommend that sponsors and trialists consider use of the FDR.

## 1 Background

Testing several experimental treatments within a single multi-arm trial is one way to dramatically increase the efficiency of drug development.^1^ Multi-arm trials reduce the sample size needed compared to separate randomised controlled trials (RCTs) due to a shared control group. They also allow a better comparison of experimental arms than a meta-analysis of separate RCTs would allow. Various non-statistical advantages exist such as reduced administrative burden of a single multi-arm trial compared to several separate trials. Multi-arm trials have been recommended in many diseases and conditions, including oncology,^2^ stroke^3^ and tuberculosis.^4^

It is a commonly accepted principle within two-arm RCTs that it is important to control the chance of incorrectly recommending ineffective experimental treatments. In hypothesis testing language this is the type I error rate, which is the chance of incorrectly rejecting a true null hypothesis. In a multi-arm trial, it is generally of interest to compare each experimental treatment against the shared control arm: this results in there being as many null hypotheses as experimental treatments.

This has caused some controversy around confirmatory multi-arm trials testing distinct interventions. Should a multi-arm trial control the chance of making any type I error, or should it be seen as equivalent to running several RCTs in parallel? Various papers^5–7^ have considered this issue and come up with different viewpoints. One point of view is that correction for multiple testing is not required. Those who argue this point out that a two-arm RCT being conducted in a particular disease would not be required to adjust its type I error rate due to the existence of other RCTs testing treatments in the same disease. Others argue that it is necessary to adjust for multiple testing. They may argue that one should be sceptical when a multi-arm trial recommends a single experimental treatment with a P-value just below .05 and that this scepticism would grow as the number of experimental arms tested in the trial increases. This factor would motivate one to control the total chance of making any type I error, known as the family-wise error rate (FWER). The disadvantage of correcting for multiple testing is that the power to recommend genuinely effective treatments will reduce, particularly when there is a large number of hypotheses.

In the phase II setting, control of the FWER can be overly conservative. This is because the selected treatments will be further tested in confirmatory phase III trials, where the final decision on whether to declare a treatment effective is made. Hence what is more appropriate is identifying a set of potentially effective treatments for further research, while allowing a controlled (low) proportion of type I errors. This can greatly increase the chance of finding truly effective treatments in phase III, while also limiting the economic and ethical cost of the additional testing of ineffective treatments.

In the phase III setting, making a type I error means that an ineffective treatment is being recommended for clinical practice. The purpose of error rate control here is thus to manage the risk of ineffective treatments being recommended for widespread use. However, if the type I error rate is controlled separately for each individual trial within a particular disease, the total chance of making at least one type I error is not controlled. This means in practice it may be acceptable to society that a proportion of treatments/interventions being recommended are actually ineffective as long as the proportion is controlled at an acceptably low level. This again motivates methods that formally control this proportion rather than the probability of making any type I error.

## 2 False Discovery Rate

The false discovery rate (FDR) is the expected proportion of rejected null hypotheses that are actually true. In the context of clinical trials, it is the proportion of selected or recommended treatments that are actually ineffective. In the case where all null hypotheses are true, the FDR is equivalent to the FWER. However when a proportion of null hypotheses are false, the FDR is less strict than the FWER: it allows some type I errors to be made as long as the number is low compared to the number of rejected null hypotheses.

The current paradigm of setting type I error rates for a trial without regard of other trials being conducted in the condition implies the FDR might be a more appropriate quantity than the FWER. In practice it would be difficult to formally control the FDR within a series of separate RCTs being run in different centres (although we consider this in section 6). It would however be possible to control this quantity within a multi-arm trial.

The advantage of the FDR is that it scales as the number of hypotheses tested increase and takes into account the proportion of treatments that are effective. If we were in the setting where a high proportion of the treatments being tested are effective, then the expected proportion of rejected hypotheses that are truly null would be low and controlling the FDR would require little downward correction to the conventional significance levels. On the other hand, if a vast majority of treatments are ineffective then the expected proportion of rejected hypotheses that are truly null is high and controlling the FDR would require a strict correction. In fact, in the situation where all null hypotheses are true, controlling the FDR also controls the FWER.

## 3 Positive and Negative Predictive Value

Type I error rate and power are undoubtedly important quantities. However, they can be somewhat misleading if we are interested in what is the chance of a recommended (ie, treatment with significant test statistic) and non-recommended treatment being truly effective or ineffective.

This justifies considering the positive predictive value (PPV) and negative predictive value (NPV). These quantities are often used to assess the utility of a diagnostic test. The main difference, compared to the power and type I error rate, is that they also take into account the proportion of hypotheses that are true or false. The PPV is the proportion of rejected null hypotheses that are truly false, or in a trial context the probability of a recommended treatment being truly effective. The NPV is the proportion of non-rejected null hypotheses that are actually true, or here the probability of a non-recommended treatment being truly ineffective. Table 1 shows how these quantities are defined in terms of the number of treatments that are effective/ineffective and recommended/not-recommended.

We note that these quantities depend on the true proportion of hypotheses that are true which would be unknown in practice. Thus, we can only ever know the PPV or NPV of a testing procedure under a given scenario where the “truth” is specified. This is similar to the type I error rate and power: these are just known under particular scenarios rather than being quantities that are estimated for a single set of observed data. It may appear that the PPV and NPV can only take a fixed number of values as they are fractions of integer values (eg, for the PPV, the number of rejected hypotheses and the number of rejected hypotheses that are actually false). However this is not the case as these integer values are random values, and the PPV or NPV of a testing procedure would be the expected value of the ratio, which can potentially take any value in the interval [0,1].

The PPV and NPV both depend on the proportion of treatments that are actually effective. A trial can have high power and reasonable type I error rate but still have a very poor PPV if very few treatments are truly effective (and conversely a poor NPV if most treatments are truly effective). As a simple example, imagine testing 100 ineffective treatments and five effective treatments. Each treatment is tested in a trial that has 5% type I error rate and 80% power. One would expect an average of five ineffective treatments to be incorrectly found to be effective, and four effective treatments to be correctly found as effective. This gives a PPV of 4/9 which is considerably lower than 80%.

In the above example, applying a multiple test correction would aim to reduce the number of ineffective treatments incorrectly found, reducing the denominator. It would also reduce the power slightly which may reduce the numerator (and denominator further) but overall it would generally lead to increases in the PPV.

## 4 Simulation Study

We conducted a simulation study to assess the PPV and NPV of multi-arm trials with different error rate correction methods.

The four methods assessed are:

1. No correction – each treatment is tested at a one-sided 2.5% significance level and recommended if its test statistic is significant;
2. FWER correction – after the results of each multi-arm trial are gathered, Dunnett’s step-down procedure^8^ is applied to the *p*-values to determine which should be rejected. This takes into account the correlation between test-statistics to increase the power compared to Holm’s procedure.^9^ This controls the FWER at 2.5%.
3. FDR correction – similarly to FWER correction, but the widely-used step-up Benjamini-Hochberg (BH) procedure^10^ is applied.
4. FDR correction using the step-down method of Somerville,^11^ that takes the correlation between the test-statistics into account to increase the power compared to the BH procedure. This procedure requires setting a minimum critical value for the test statistics.

Both methods 3 and 4 are set to control the FDR at 2.5%.

The values we varied in the simulation study were the number of experimental arms, K ∈ {3, 5, 10}, and the probability of each experimental treatment being effective, p ∈ {0.05,0.06,…, 0.94,0.95}.

The simulation study assumes that K experimental treatments are compared against a shared control arm (with equal allocation to each arm). The K null hypotheses tested are:

$$H_{1}:\delta_{1}\leq 0,H_{2}:\delta_{2}\leq 0,\ldots ,H_{K}:\delta_{K}\leq 0,$$

where δ_j_ represents the treatment effect of the j-th experimental treatment against control (positive values represent the new treatment being better than control). Each hypothesis, H_j_, is tested with associated standard test statistic Z_j_.

For each combination of K and p we conducted 100 000 simulation replicates. Each replicate followed the following process:

1. Simulate the number of treatments in the trial that are truly effective from a Binomial (K,p) distribution. The remaining treatments are set to be ineffective.
2. For each effective treatment, set the mean of the test statistic to give a marginal power of 90% (ie, a value m such that P(Z - m > Φ^-1^(1 − α)) = 0.9, where Z is a standard normal variable and α is 0.025 or 0.1). For ineffective treatments, set the mean of the test statistic to be 0.
3. Set the covariance matrix for test statistics to have diagonal entries of 1 and non-diagonal entries of 0.5 to represent a multi-arm trial with shared control and equal sample size per arm.
4. Simulate a realization of a multivariate normal random variable with mean vector consisting of the means in step 2 and covariance matrix set in step 3. This represents the vector of test statistics for each arm vs control.
5. Find marginal one-sided P-values from each test statistic by comparing them to the standard normal distribution.
6. Record which hypotheses were rejected after (a) no correction; (b) the Dunnett step-down method is applied; (c) Benjamini-Hochberg is applied; (d) the step-down FDR correction of Somerville is applied (with a minimum critical value for the test statistics set equal to Φ^-1^(1 − α)).

For each replicate, the PPV can be estimated as the proportion of rejected hypotheses that were in fact associated with effective treatments. If no hypotheses were rejected, that replicate was excluded from consideration. Similarly, for the NPV, the proportion of non-rejected hypotheses that were in fact associated with non-effective treatments was estimated. If all hypotheses were rejected, the replicate was excluded from consideration. The PPV and NPV for each correction method was then estimated as the average across all non-excluded replicates.

Excluding replicates in which all or none (for the NPV and PPV respectively) of the hypotheses were rejected gives an estimate of the average properties of individual trials, conditional on the metric being relevant. For example, if a trial rejects no hypotheses then the PPV is arguably not relevant for interpreting the results of the trial. In Section 2 of the Supporting Information, we present results that consider a more global measure of the properties (ie, if all trials used such an approach, what the properties would be across all trials). We sum the number of correctly rejected and the number of rejected hypotheses across all replicates; the PPV is estimated as the ratio. Similarly the NPV is estimated as the ratio as the total number of correctly non-rejected and non-rejected hypotheses.

## 5 Results

**Figure 1:**
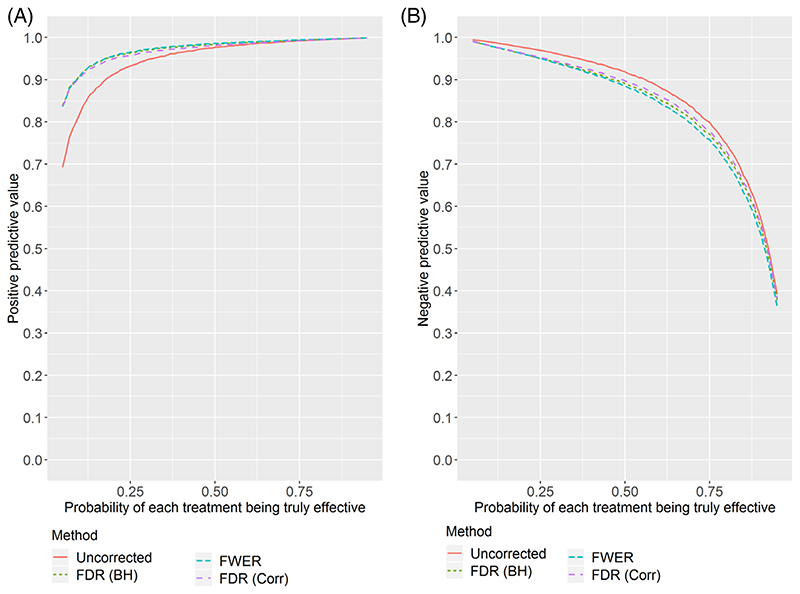
Positive and negative predictive values estimated from the simulation study with three experimental arms as the proportion of treatments which are truly effective changes. A, Positive predictive value. B, Negative predictive value

**Figure 2:**
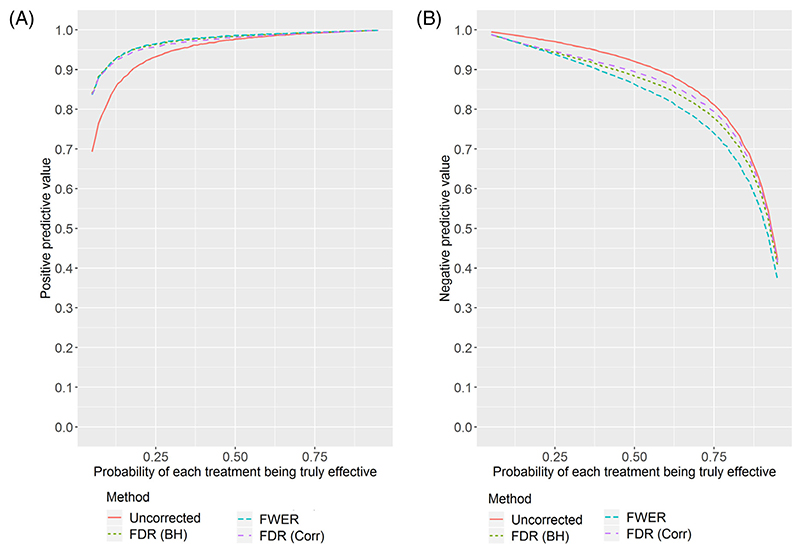
Positive and negative predictive values estimated from the simulation study with five experimental arms as the proportion of treatments which are truly effective changes. A, Positive predictive value. B, Negative predictive value

Figures 1 and 2 show (respectively for *K* = 3 and *K* = 5) the estimated PPV and NPV for the four correction methods as the true probability of each experimental treatment being effective varies. The simulation study shows some interesting results.

First, the PPV can be low in the setting where few treatments are truly effective. With the uncorrected method, despite a marginal power of 90%, there is a lower than 90% chance that a recommended treatment is truly effective if the true proportion of effective treatments is below 1/3. This may cause concern in some disease areas where few treatments are found to be effective. Adjusting for multiple testing results in much higher PPV in the low proportion setting, with there being little difference between FWER and FDR.

On the other hand, the NPV can be notably reduced by applying multiple testing correction, with the FDR correction losing less than the FWER correction. This is especially the case for five experimental arms, and when most treatments are truly effective. In Figure S1, we show the results for 10 experimental arms, where the differences are even more pronounced.

Table 2 shows the PPV and NPV for a selection of points on the figures. It also shows the average FWER across replicates. The FWER is well-controlled at the nominal level by the step-down Dunnett procedure. When applying the step-down FDR procedure, the FWER is above 0.025 in some cases; the inflation is moderate when the number of arms is large and the proportion of effective arms is around 0.5. Not applying a multiple testing correction results in high FWER when the number of arms is large and the proportion of effective arms is low.

To emulate a phase II trial setting, we also considered a more relaxed error rate of 0.1. Figures S2-S4 shows analogous plots for K = 3, 5, 10 in this setting. The conclusions are unchanged, although the methods are somewhat more separated for the NPV and PPV.

Section 4 of the Supporting Information presents and discusses results for when the number of effective arms is assumed to be fixed in each simulation replicate, rather than being a random variable.

## 6 Discussion

The results we present here indicate that FDR correction offers an appealing compromise. In the low efficacy setting, it avoids the loss of PPV from not correcting; in the high efficacy setting it avoids the loss of NPV from FWER correction. There is little noticeable difference between the different methods when the number of experimental arms is three - most differences only become apparent when the number of experimental arms is five or above. For all the methods considered, raw *P*-values can be reported as normal, with the procedure used to denote which hypotheses are rejected. Alternatively, adjusted *P*-values can straightforwardly be produced that are compared to the nominal alpha level (apart from the Somerville approach).

We should emphasise that the Dunnett step-down approach we use for FWER control is, in some ways, not analogous to the Benjamini-Hochberg approach we use for FDR control. The former takes into account the known correlation between test statistics (from the shared control group) and picks the critical value appropriately; the latter does not. The approach proposed by Somerville^11^ does use a known covariance structure like Dunnett does for FWER control (but has not been rigorously proved to control the FDR). As the simulation results show, this further improves the NPV, although at the cost of a slight reduction in the PPV.

We have only considered methods that do not require specification of the proportion of hypotheses that are true. If the proportion of hypotheses that are true could be specified, or estimated from the data, then other approaches for FDR control exist that improve on the power of the original Benjamini-Hochberg method,^12^ although they assume independence of the test statistics. In particular, if the proportion of hypotheses that are true is known (and equal to π_0_), then the BH procedure (and others) can be applied at level α/π_0_ instead. However this raises the difficult issue of how to get a sensible figure to use for the proportion of true null hypotheses. One could consider the proportion of treatments tested in the broad clinical area that were found to be effective (eg, by looking at published work on trial success rates such as^13^). However, this is not necessarily the correct figure due to type I and II errors made. Also, there is no guarantee that the treatments being tested in the current trial are representative of other treatments tested in the same area. Clearly this is an issue that requires further thought.

Considering the objective of the trial may provide insight into which correction method is most relevant. If the aim is to identify the best treatment (or a single treatment to proceed with), then the FWER may be more appropriate. If for example only a relatively small proportion of treatments are effective, then making even a single type I error likely means that the overall conclusion of the trial (declaring a single treatment effective) will also be in error. If finding multiple effective experimental treatments is of interest, particularly in a setting where there is (believed to be) a relatively high proportion of effective treatments, then the FDR may be more appropriate. In particular, using the FDR in this setting can result in a substantially higher conjunctive power (the chance of correctly finding that all truly effective treatments are effective). The FDR may also be more appropriate in phase II trials, given that the identified effective treatments will then be tested in a confirmatory phase III study with strong FWER control. The Supporting Information provides all results with a relaxed error rate of 0.1 instead of 0.025 used here.

In pharmaceutical trials it is important to consider regulatory issues. As already mentioned, strong control of the FWER is expected by regulators in order to make a confirmatory claim. In practice however, regulatory approval from the US FDA traditionally requires at least two statistically significant confirmatory trial results (although 37% of FDA approvals from 2005-12 were based on only a single trial^14^), which we have not considered. This will mean the PPV is likely to be much higher for these phase III trial settings, although the NPV may also be considerably lower. Hence our results from a phase II setting (with a relaxed alpha) may be more relevant to industry trials in practice.

Although we have considered only multi-arm trials with distinct treatment arms, FDR may be relevant in other settings. For example in basket trials (which test one drug in distinct patient subgroups, usually different tumour types in which a tumour mutation is present) it has been recommended to strongly control the FWER.^15^

Including adaptive designs within multi-arm trials is popular, with many different approaches having been considered (eg, ^16–18^). Most methodological papers have either not considered correction or considered FWER correction. Work from genomics^19,20^ provides methods for controlling the FDR within a multi-stage design. This would be a useful area to extend in the future.

A related issue is that we have assumed that all the treatments in a multi-arm trial are available for testing at once. However, this is not the case for platform trials,^21^ which drop treatments from the trial after they have been formally tested for effectiveness and allow new treatment arms to be added to the trial as it progresses. In such a setting with sequential testing of hypotheses, so-called online error rate control can be applied to control either the FDR^22^ or the FWER.^23^

Finally, even in the challenging setting of formally controlling the FDR for a series of separate RCTs being run in different centres, the previously mentioned methodological research^22–24^ could provide (at least theoretically) a way of controlling the FDR.

## Supplementary Material

Supplementary File

## Figures and Tables

**Table 1:** Summary of error rate and predictive values in terms of number of treatments within each category (recommended/non-recommended vs truly effective/truly ineffective)

	Recommended treatments	Non-recommended treatments
Non-effective treatments	A	B
Effective treatments	C	D
Family-wise error rate	P(A > 0)	
FDR	E(A/[A + C])^a^	
PPV	C/(A + C)	
NPV	B/(B + D)	

Abbreviations: FDR, false-discovery rate; NPV, negative predictive value; PPV, positive predictive value.

a The FDR is defined as 0 when the denominator A + C is 0.

**Table 2:** Statistical properties of different correction techniques for a selected number of values for the probability of each treatment being effective

		PPV			NPV			FWER		
*K*	*P*	Uncorrected	FWER control	FDR control (Corr)	Uncorrected	FWER control	FDR control (Corr)	Uncorrected	FWER control	FDR control (Corr)
3	.05	0.687	0.834	0.833	0.995	0.991	0.990	0.060	0.025	0.025
3	.20	0.912	0.956	0.949	0.976	0.961	0.962	0.052	0.024	0.031
3	.50	0.977	0.986	0.981	0.918	0.886	0.897	0.034	0.022	0.029
3	.80	0.994	0.995	0.994	0.747	0.705	0.728	0.015	0.013	0.015
3	.95	0.999	0.999	0.999	0.392	0.361	0.380	0.004	0.004	0.004
5	.05	0.709	0.884	0.883	0.995	0.988	0.988	0.089	0.026	0.027
5	.20	0.921	0.973	0.961	0.976	0.951	0.955	0.076	0.024	0.038
5	.50	0.977	0.990	0.982	0.922	0.864	0.896	0.052	0.023	0.044
5	.80	0.994	0.996	0.994	0.768	0.695	0.753	0.024	0.017	0.024
5	.95	0.999	0.999	0.999	0.423	0.371	0.416	0.006	0.005	0.006
10	.05	0.747	0.935	0.932	0.995	0.985	0.984	0.140	0.025	0.027
10	.20	0.927	0.984	0.967	0.977	0.937	0.947	0.126	0.026	0.055
10	.50	0.978	0.994	0.981	0.922	0.823	0.890	0.089	0.025	0.076
10	.80	0.994	0.997	0.994	0.788	0.659	0.776	0.043	0.022	0.043
10	.95	0.999	0.999	0.999	0.474	0.378	0.470	0.012	0.010	0.012

Abbreviations: FDR, false-discovery rate; FWER, family-wise error rate; K, number of experimental arms; NPV, negative predictive value; P, probability of each experimental arm being effective; PPV, positive predictive value.

## Data Availability

Data sharing is not applicable to this article as no new data were created or analyzed in this study.
