# Supplementary material for: Controlling type I error rates in multi-arm clinical trials: A case for the false discovery rate
Source: Pharm Stat. Author manuscript; Available in PMC 2022 Jan 5. (PMC7612170; doi:10.1002/pst.2059)
Supplement: Supplementary File [file EMS140641-supplement-Supplementary_File.docx]

**Supplementary material for “Controlling type I error rates in multi-arm clinical trials: a case for the false discovery rate”**

**Supplementary results**

1. PPV and NPV for K=10

Figure S1: positive and negative predictive values estimated from the simulation study with ten experimental arms as the proportion of treatments which are truly effective changes. Type I error rate/FDR/FWER controlled at 0.025 (one-sided) by the various approaches.

1. Positive predictive value


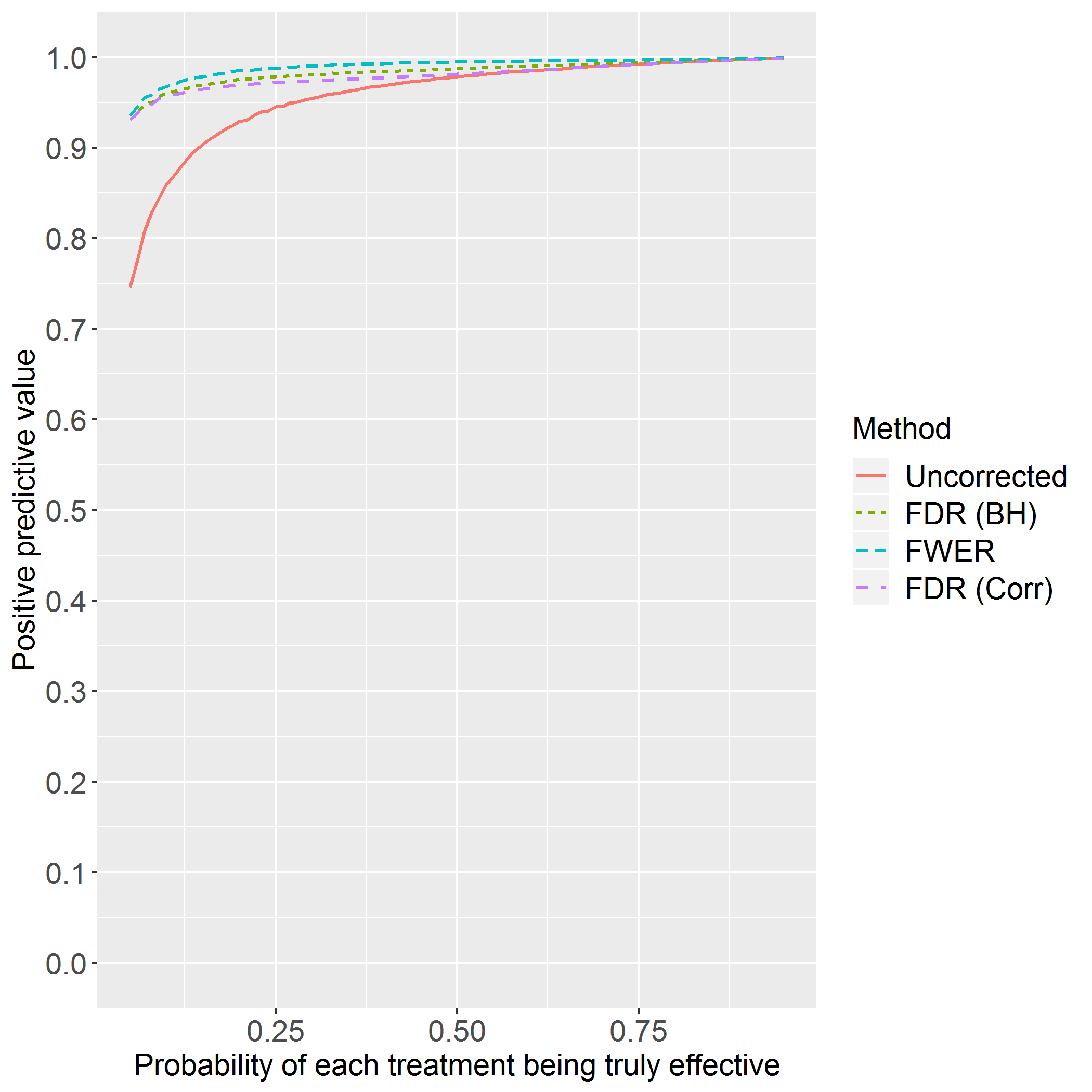


1. Negative predictive value


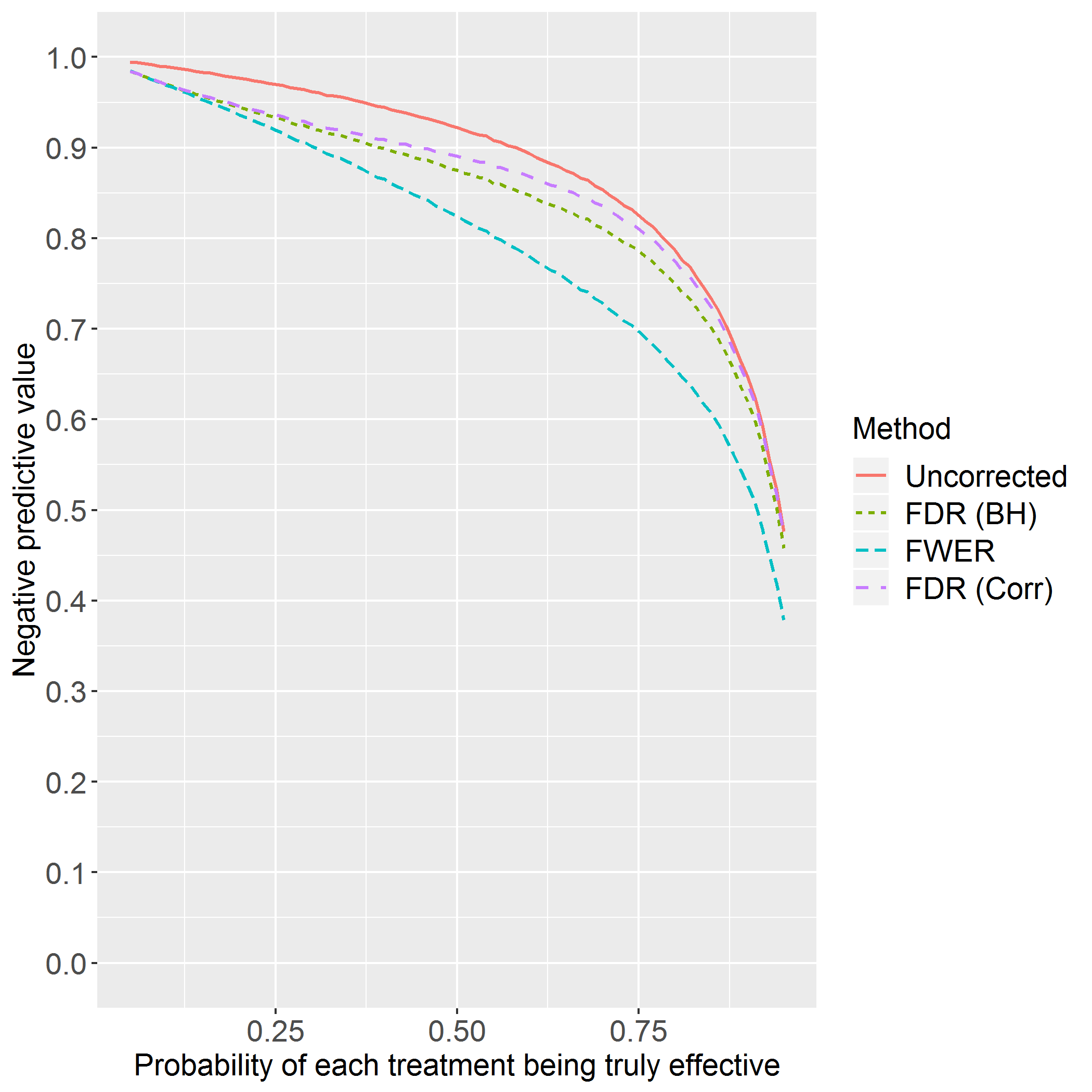


1. Results for error rates controlled at 0.1

Figure S2: positive and negative predictive values estimated from the simulation study with three experimental arms as the proportion of treatments which are truly effective changes. Type I error rate/FDR/FWER controlled at 0.1 (one-sided) by the various approaches.

1. Positive predictive value


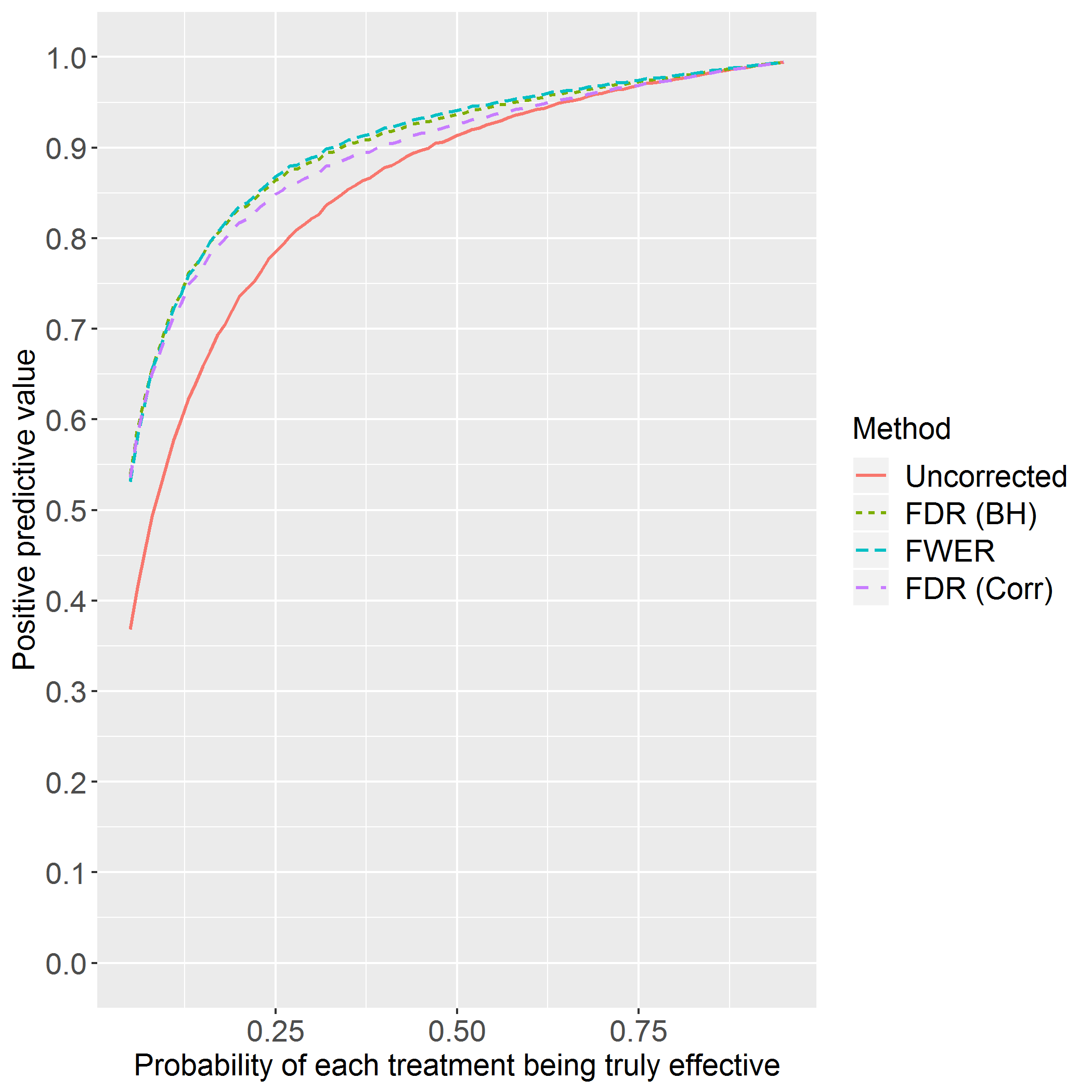


1. Negative predictive value


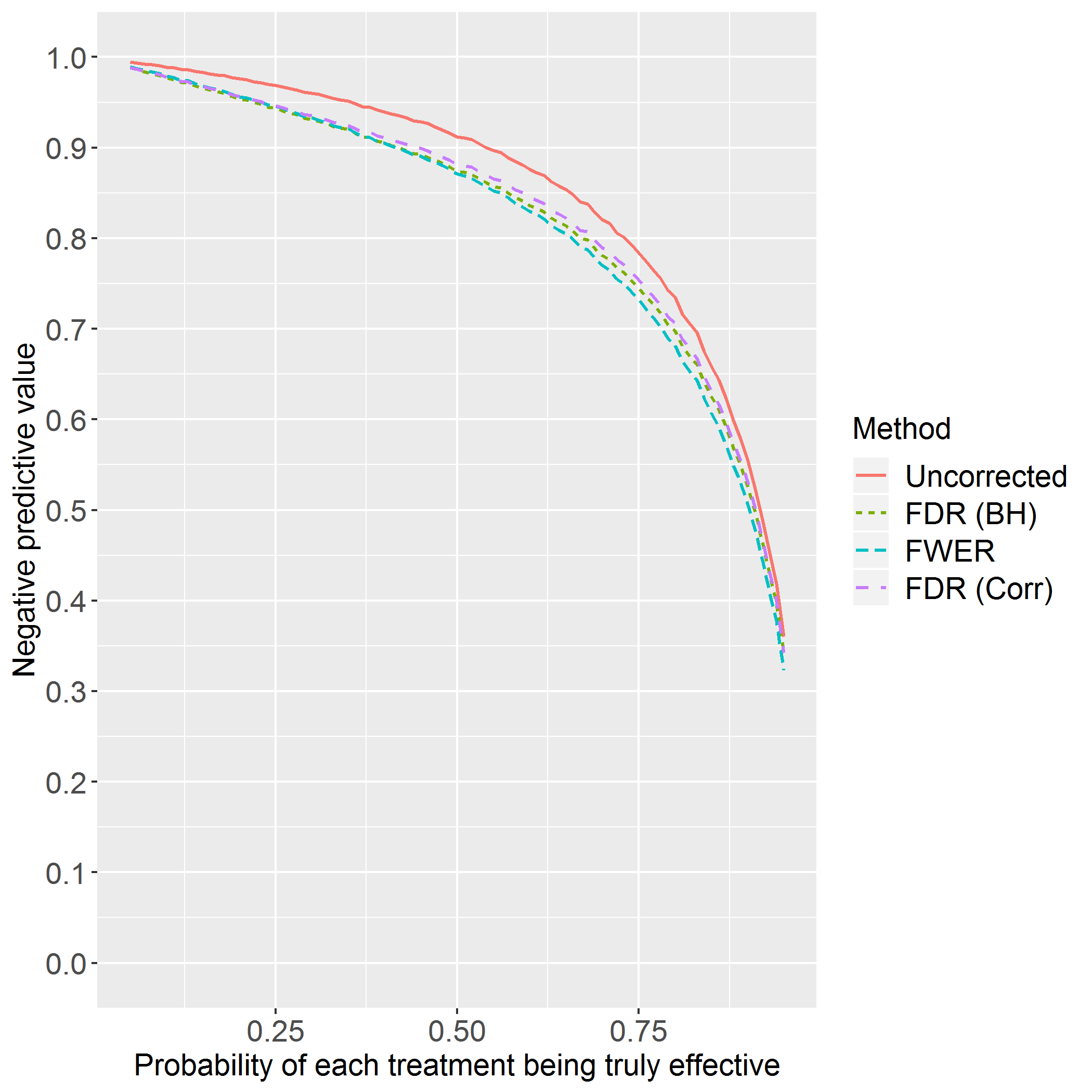


Figure S3: positive and negative predictive values estimated from the simulation study with five experimental arms as the proportion of treatments which are truly effective changes. Type I error rate/FDR/FWER controlled at 0.1 (one-sided) by the various approaches.

1. Positive predictive value


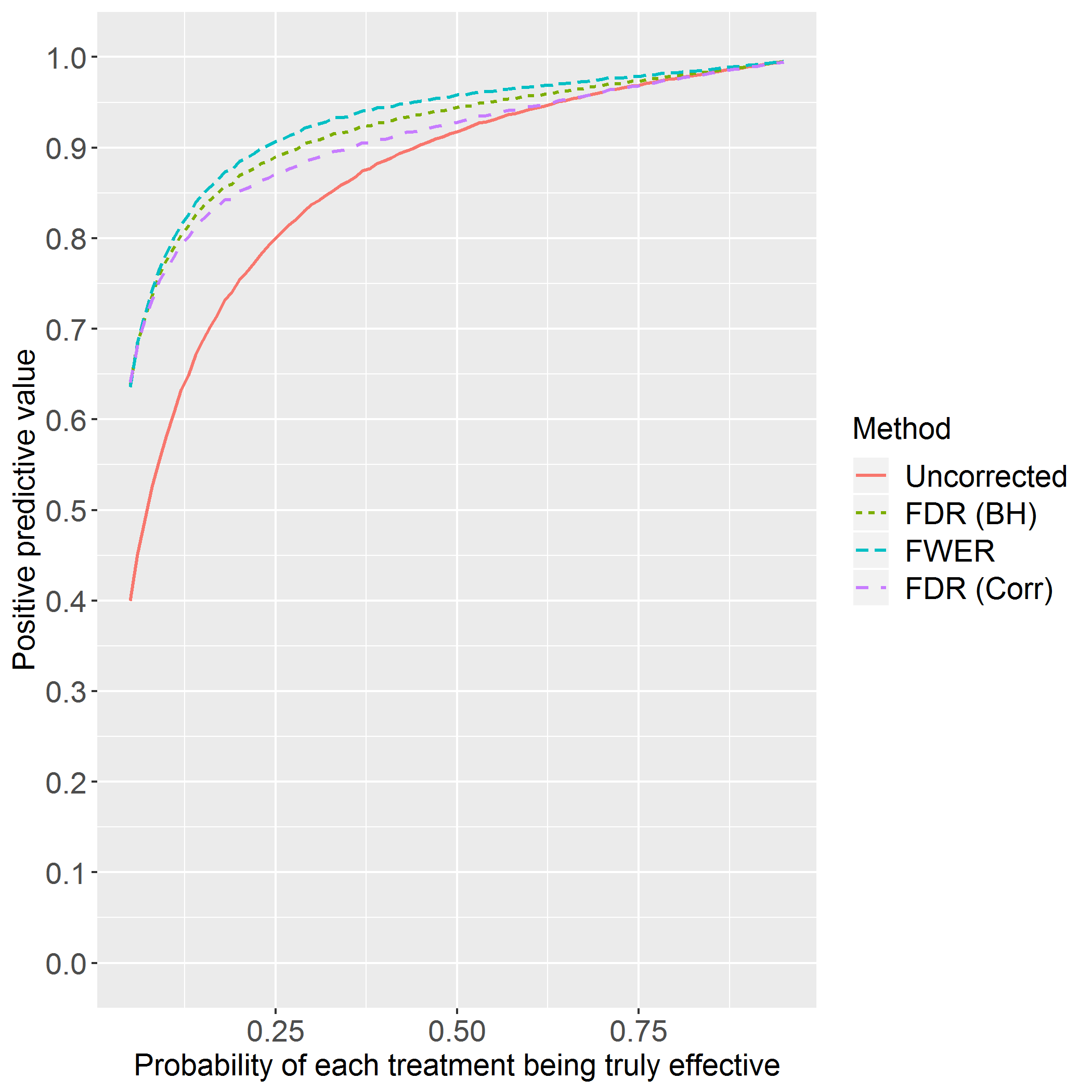


1. Negative predictive value


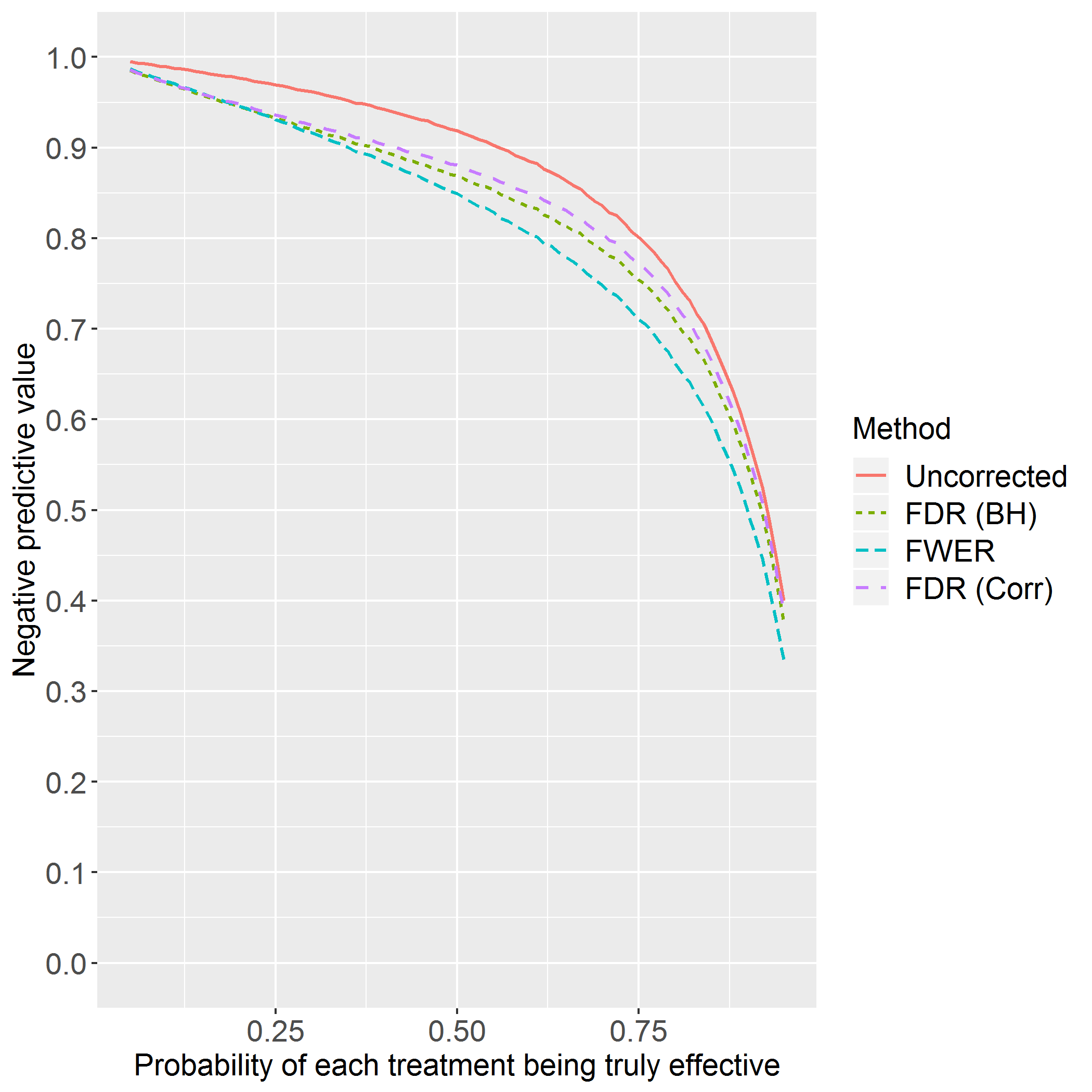


Figure S4: positive and negative predictive values estimated from the simulation study with ten experimental arms as the proportion of treatments which are truly effective changes. Type I error rate/FDR/FWER controlled at 0.1 (one-sided) by the various approaches.

1. Positive predictive value


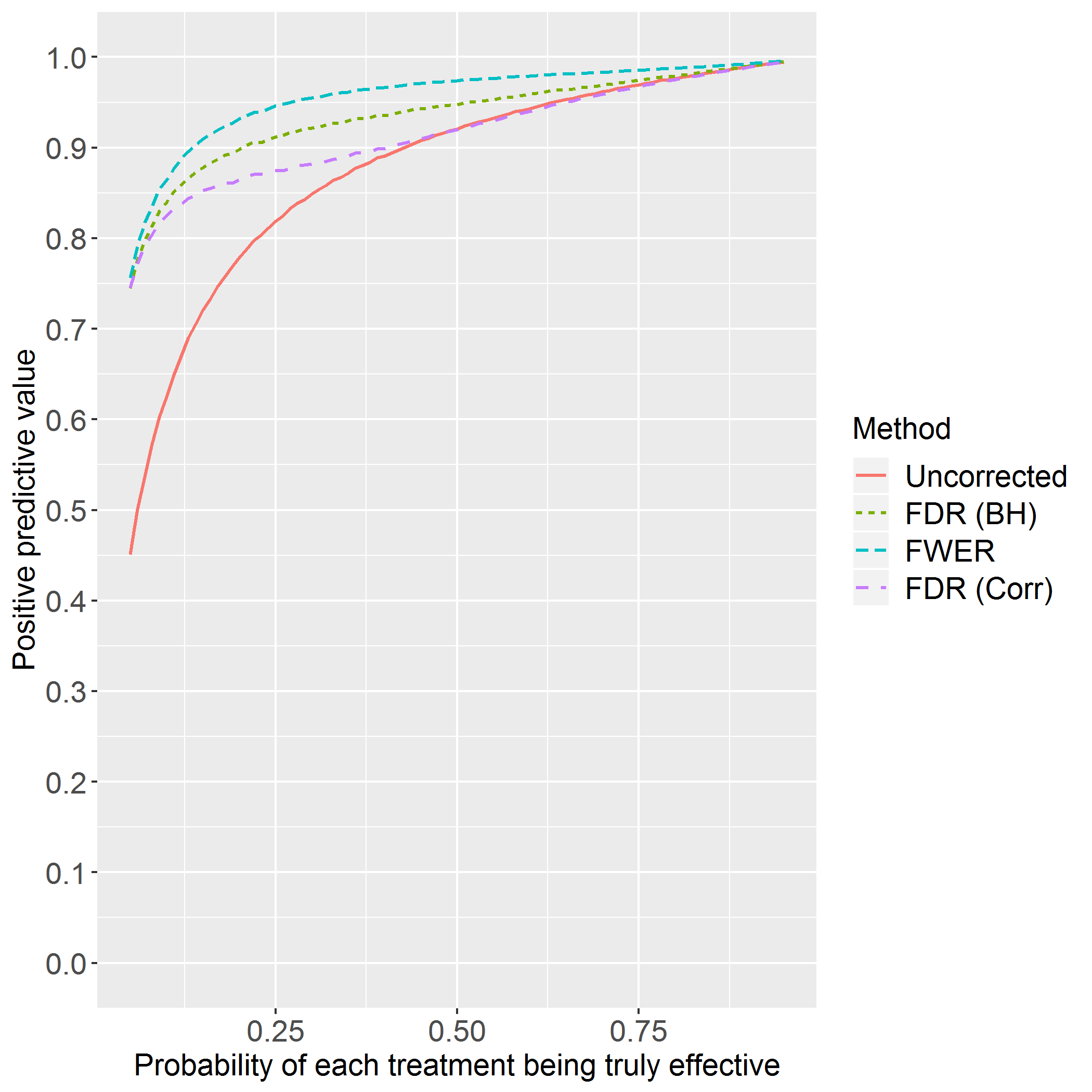


1. Negative predictive value


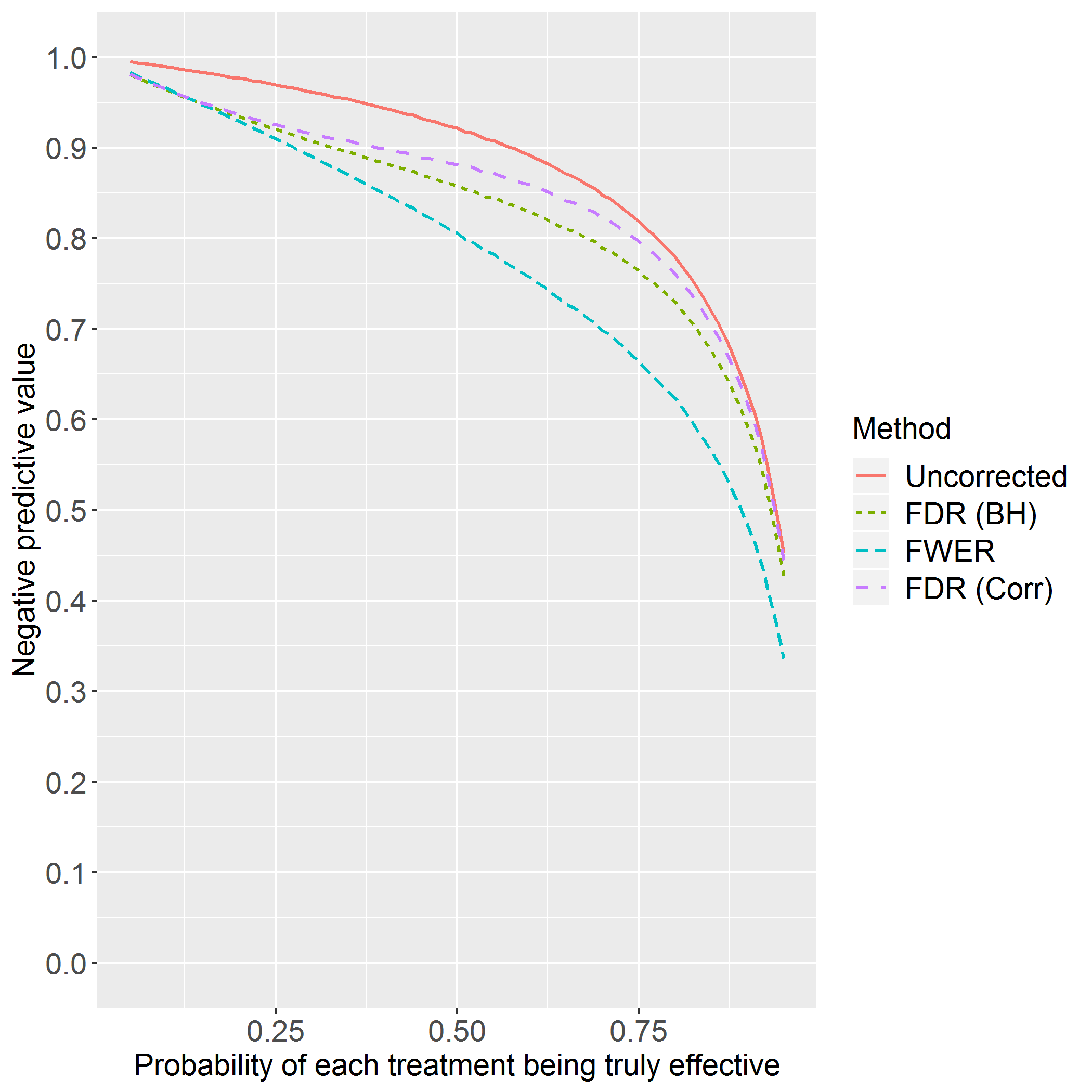


Table S1 –properties of different correction methods for a selected number of values for the probability of each treatment being effective, error rate set to 0.1. K – number of experimental arms; p – probability of each experimental arm being effective; PPV – positive predictive value; NPV – negative predictive value; FWER – family-wise error rate.

|  |  | **PPV** | | | **NPV** | | | **FWER** | | |
| --- | --- | --- | --- | --- | --- | --- | --- | --- | --- | --- |
| **K** | **p** | **Uncorrected** | **FWER control** | **FDR control (Corr)** | **Uncorrected** | **FWER control** | **FDR control (Corr)** | **Uncorrected** | **FWER control** | **FDR control (Corr)** |
| 3 | 0.05 | 0.364 | 0.525 | 0.533 | 0.995 | 0.990 | 0.989 | 0.211 | 0.102 | 0.096 |
| 3 | 0.20 | 0.737 | 0.836 | 0.819 | 0.976 | 0.957 | 0.956 | 0.182 | 0.097 | 0.113 |
| 3 | 0.50 | 0.915 | 0.942 | 0.927 | 0.913 | 0.872 | 0.883 | 0.125 | 0.086 | 0.108 |
| 3 | 0.80 | 0.976 | 0.980 | 0.976 | 0.730 | 0.678 | 0.702 | 0.056 | 0.047 | 0.054 |
| 3 | 0.95 | 0.995 | 0.995 | 0.995 | 0.366 | 0.329 | 0.349 | 0.015 | 0.014 | 0.014 |
| 5 | 0.05 | 0.399 | 0.640 | 0.645 | 0.995 | 0.987 | 0.985 | 0.285 | 0.098 | 0.092 |
| 5 | 0.20 | 0.754 | 0.885 | 0.850 | 0.977 | 0.946 | 0.947 | 0.256 | 0.099 | 0.135 |
| 5 | 0.50 | 0.918 | 0.959 | 0.930 | 0.919 | 0.849 | 0.880 | 0.185 | 0.094 | 0.156 |
| 5 | 0.80 | 0.977 | 0.983 | 0.976 | 0.752 | 0.662 | 0.727 | 0.088 | 0.065 | 0.086 |
| 5 | 0.95 | 0.995 | 0.995 | 0.994 | 0.401 | 0.336 | 0.388 | 0.024 | 0.022 | 0.024 |
| 10 | 0.05 | 0.452 | 0.761 | 0.750 | 0.995 | 0.983 | 0.981 | 0.396 | 0.099 | 0.094 |
| 10 | 0.20 | 0.778 | 0.931 | 0.865 | 0.977 | 0.929 | 0.937 | 0.366 | 0.099 | 0.188 |
| 10 | 0.50 | 0.921 | 0.974 | 0.921 | 0.921 | 0.805 | 0.881 | 0.283 | 0.096 | 0.263 |
| 10 | 0.80 | 0.976 | 0.987 | 0.975 | 0.778 | 0.621 | 0.759 | 0.155 | 0.085 | 0.154 |
| 10 | 0.95 | 0.995 | 0.996 | 0.994 | 0.453 | 0.336 | 0.446 | 0.047 | 0.037 | 0.047 |

1. Results when PPV and NPV estimated by summing across replicates

As described in the main paper, for replicates where no hypotheses were rejected, it is not clear how to estimate the PPV. Similarly, for replicates where all hypotheses were rejected, it is not clear how to estimate the NPV. Such replicates can be common for some configurations (e.g. when *K*=3 for low or high proportions of treatments being effective respectively).

In the main results, these replicates were excluded from consideration. We believe this is justifiable when considering trial-level properties: it is not a well-defined problem to consider the chances of rejected hypotheses corresponding to truly effective treatments when no hypotheses were rejected (similarly for when all hypotheses were rejected, the chances of the non-rejected hypotheses corresponding to non-effective treatments).

It is also relevant to consider the properties of trials from a more societal level. That is, if different multiple testing procedures were adopted, then what would the long run properties of trials be.

For this we repeated the simulation study from the main paper with one main difference. Instead of calculating the PPV and NPV for each replicate, we estimated it by summing across all replicates. For example, the PPV was estimated by summing the number of correctely rejected null hypotheses and the number of rejected null hypotheses across all replicates and then estimating the PPV as the ratio of these. In this section we provide all figures and tables using this approach.

**Figure S5.** Positive and negative predictive values estimated from the simulation study with three experimental arms as the proportion of treatments which are truly effective changes. Type I error rate/FDR/FWER controlled at 0.1 (one-sided) by the various approaches.

1. Positive predictive value


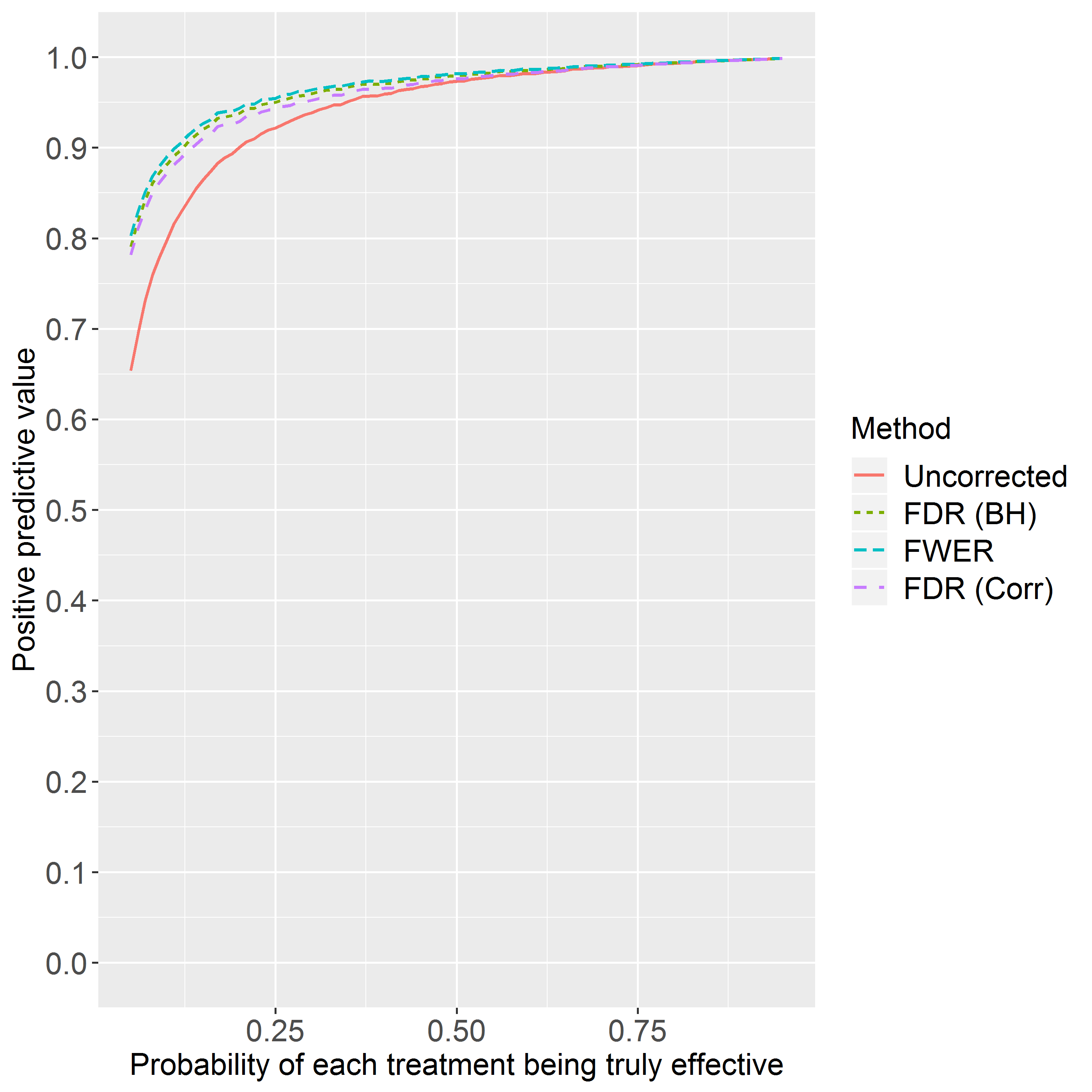


1. Negative predictive value


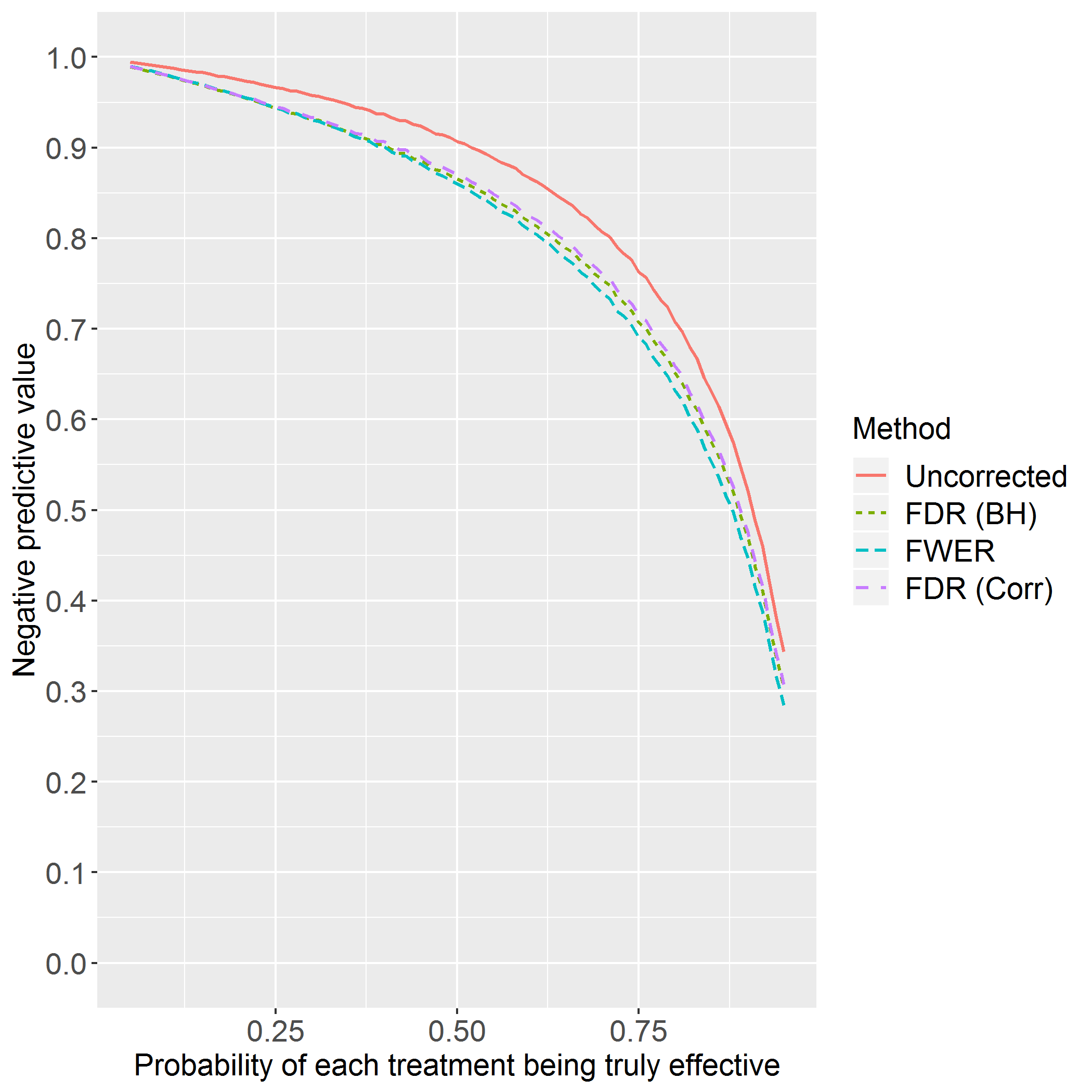


**Figure S6.** Positive and negative predictive values estimated from the simulation study with five experimental arms as the proportion of treatments which are truly effective changes. Type I error rate/FDR/FWER controlled at 0.1 (one-sided) by the various approaches.

1. Positive predictive value


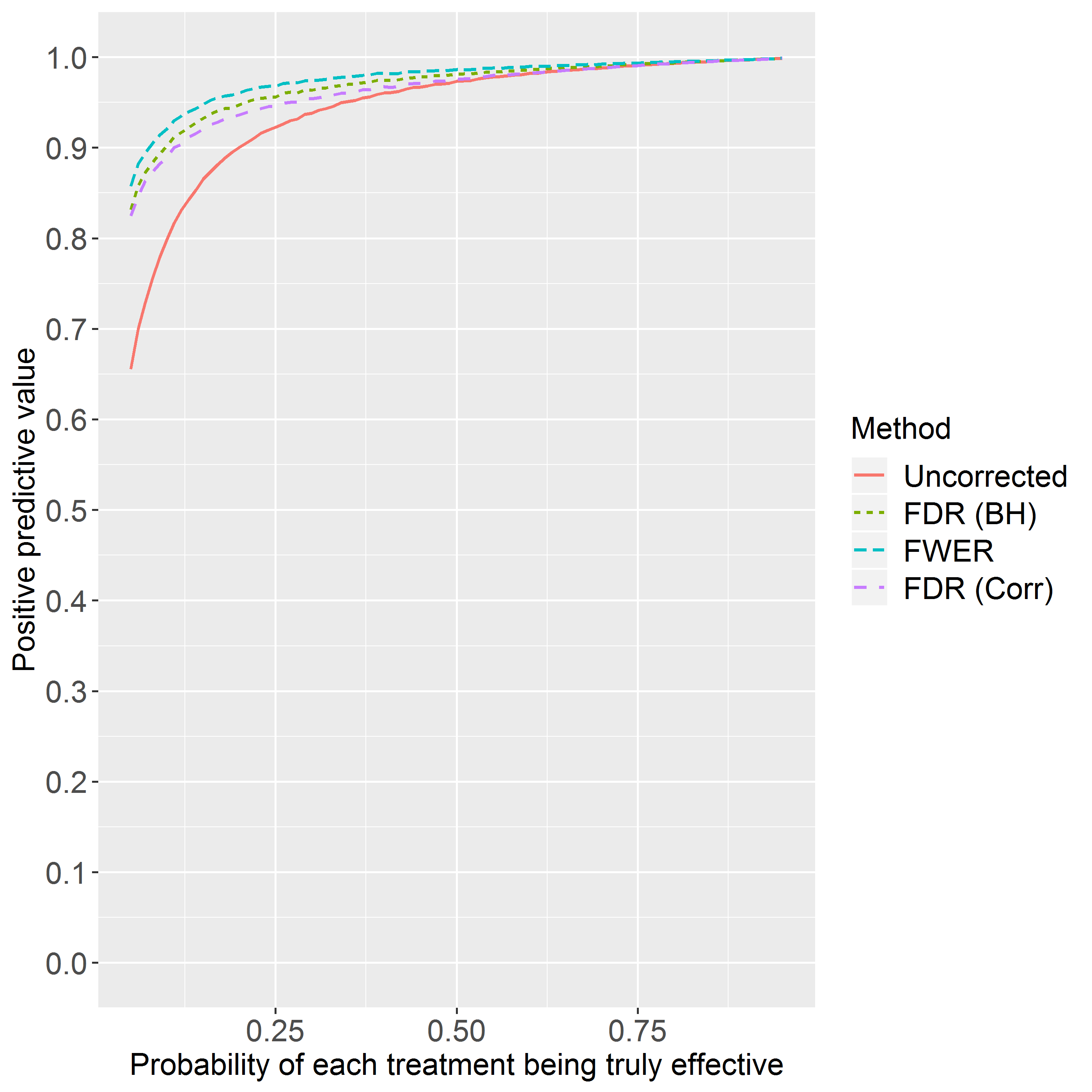


1. Negative predictive value


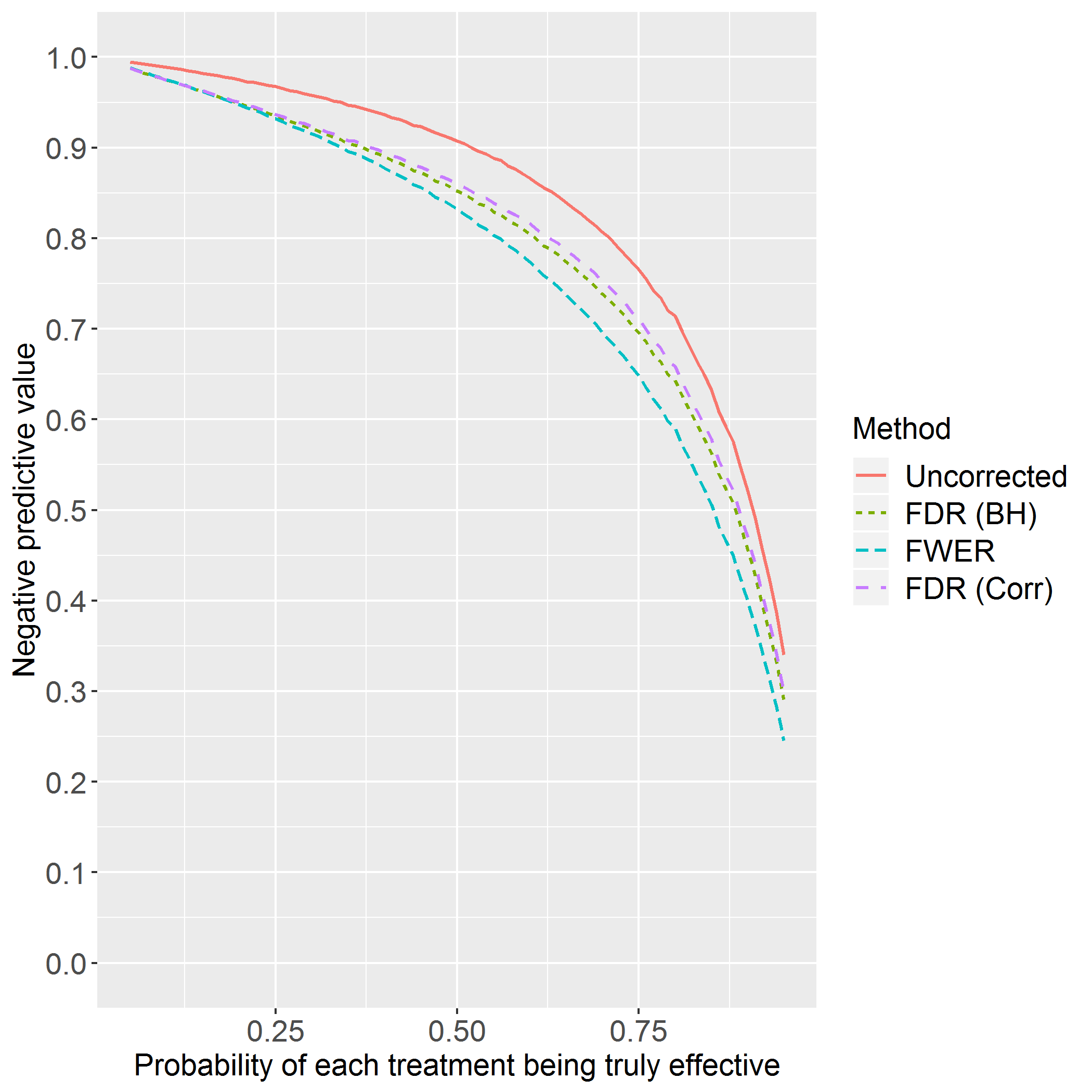


**Figure S7**: positive and negative predictive values estimated from the simulation study with ten experimental arms as the proportion of treatments which are truly effective changes. Type I error rate/FDR/FWER controlled at 0.025 (one-sided) by the various approaches.

1. Positive predictive value


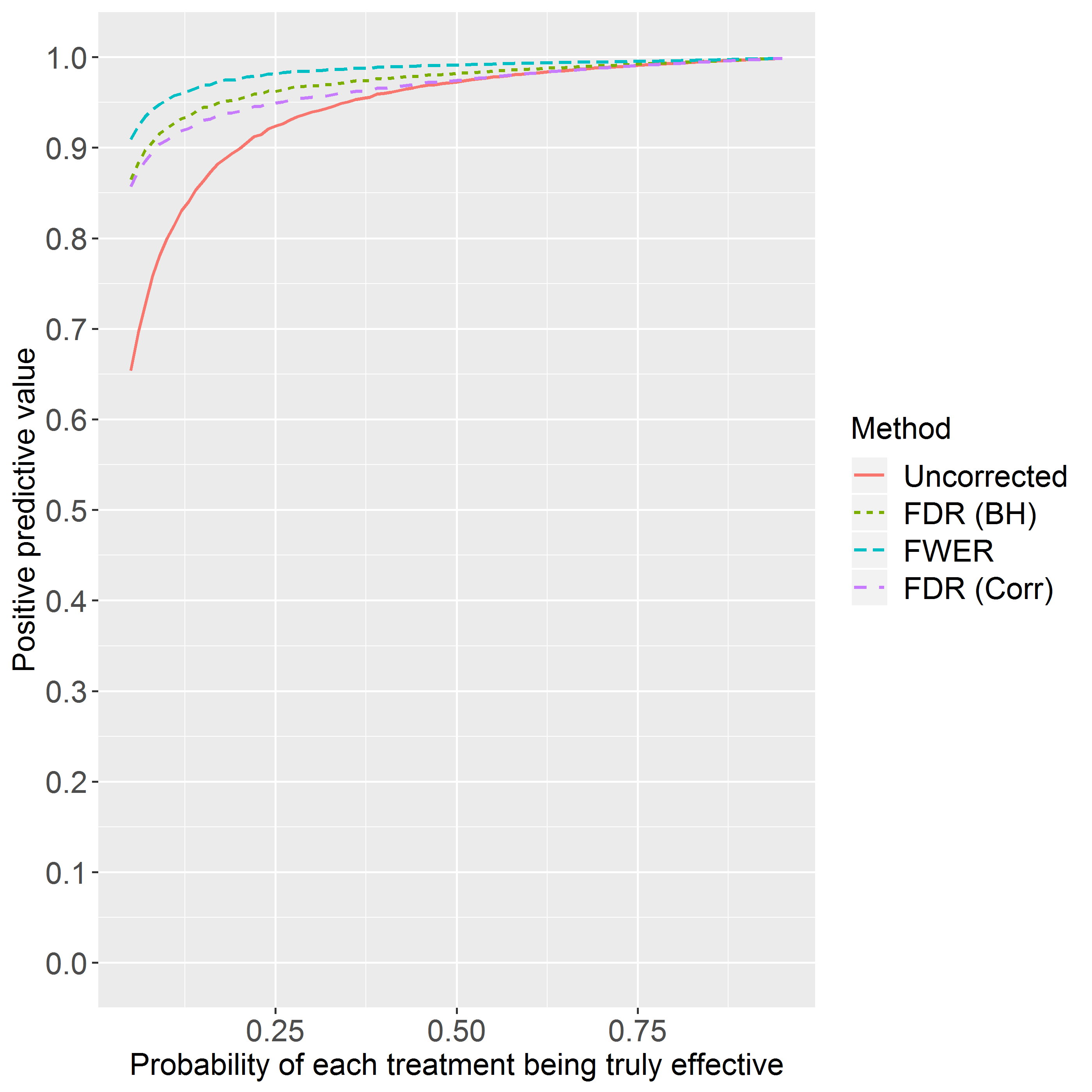


1. Negative predictive value


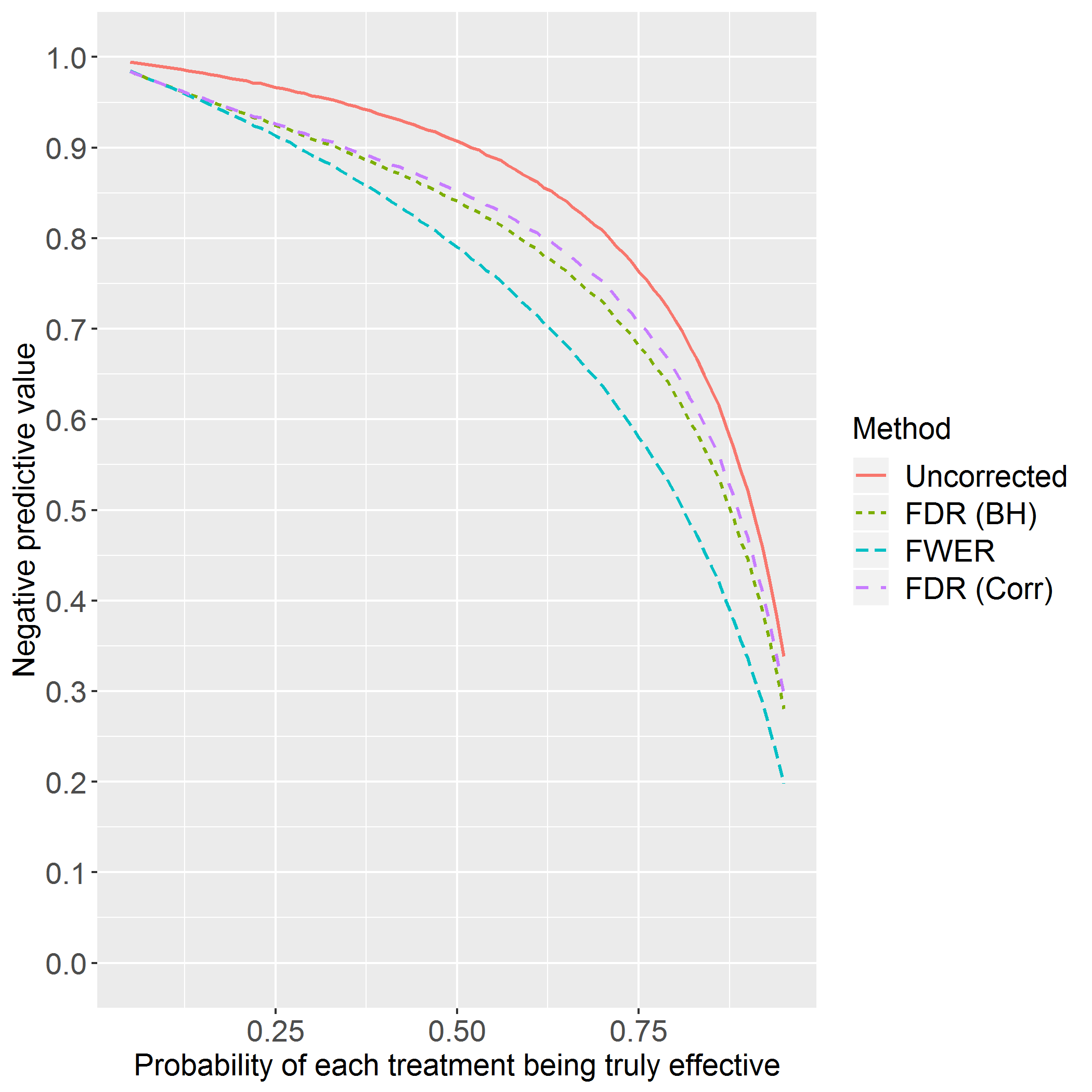


**Figure S8**: positive and negative predictive values estimated from the simulation study with three experimental arms as the proportion of treatments which are truly effective changes. Type I error rate/FDR/FWER controlled at 0.1 (one-sided) by the various approaches.

1. Positive predictive value


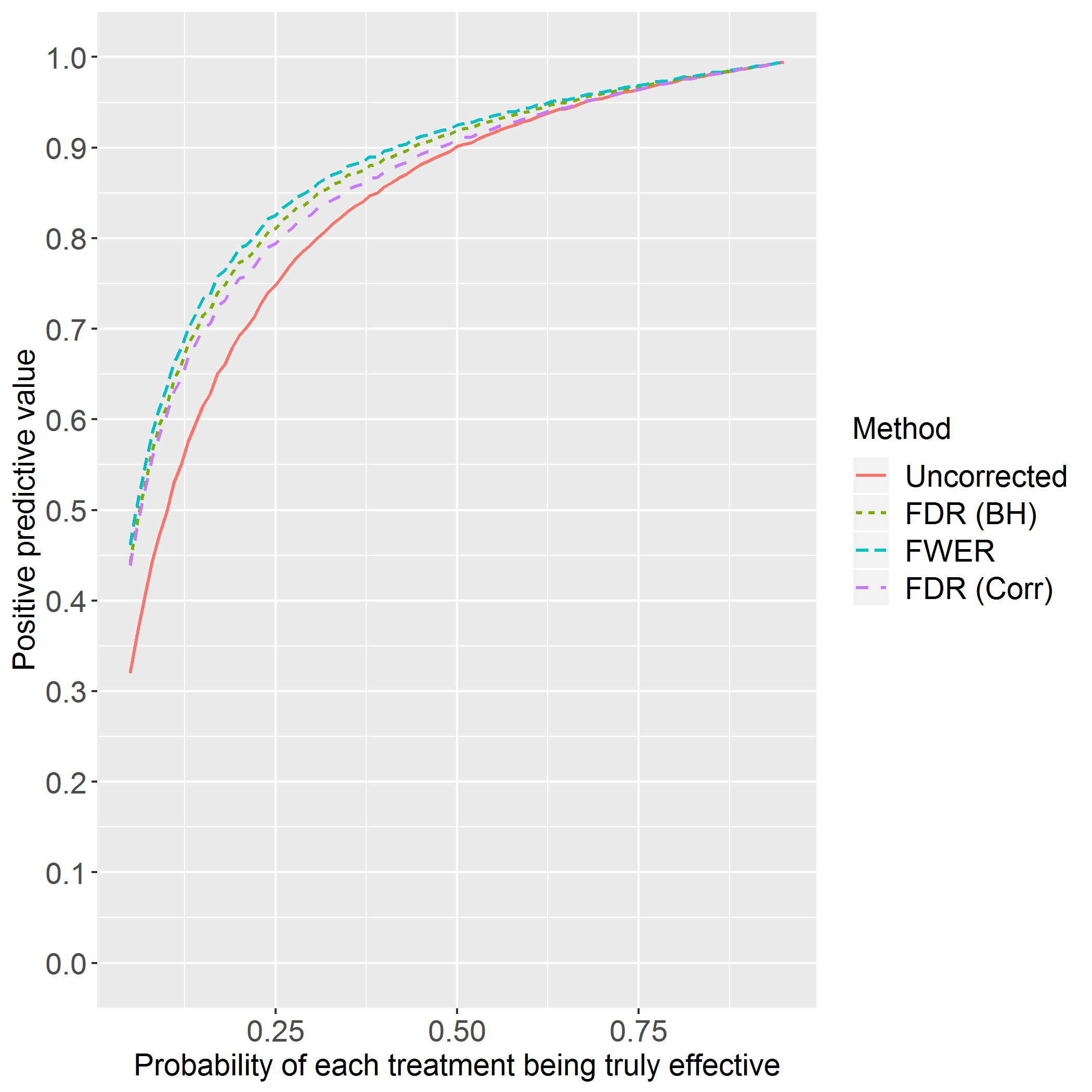


1. Negative predictive value


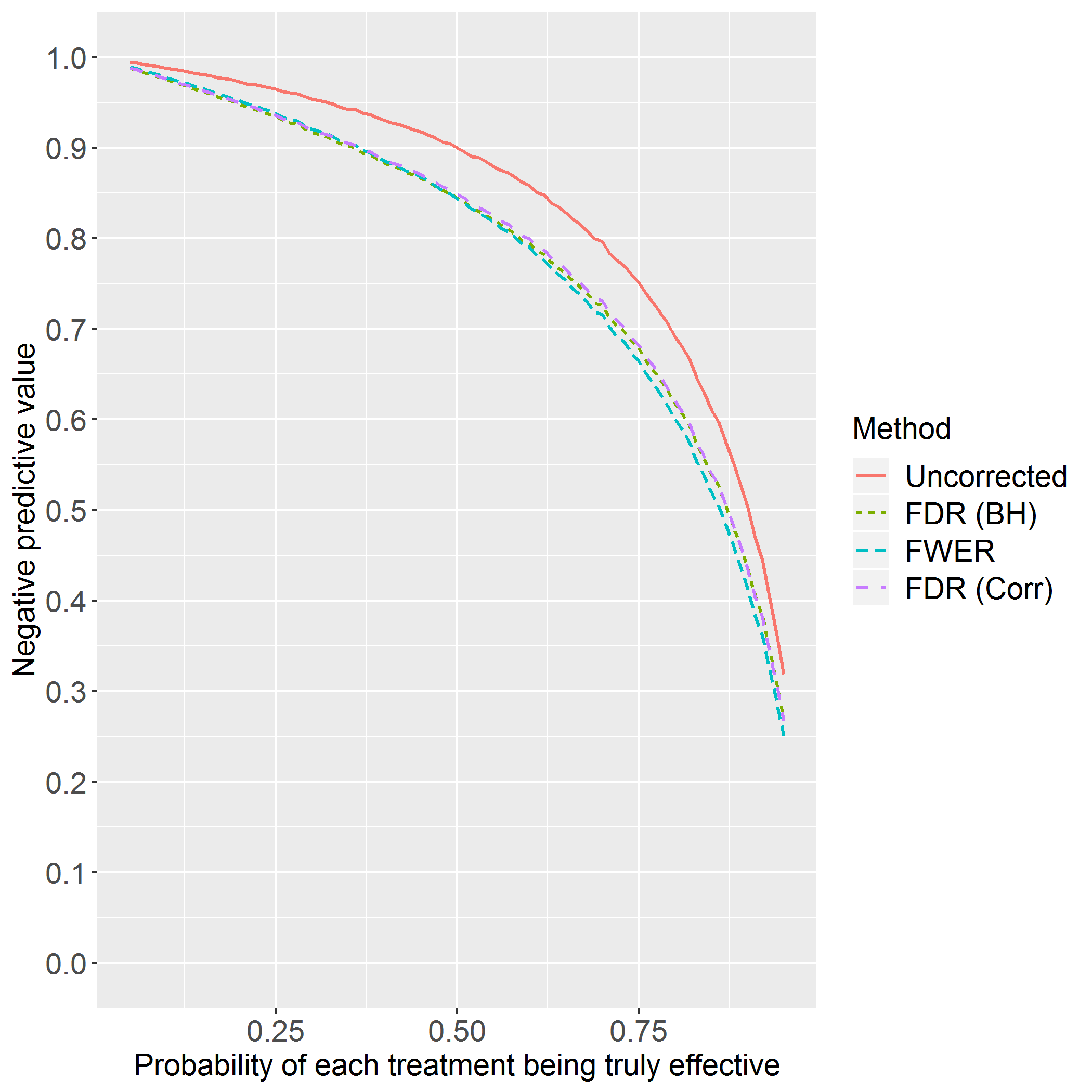


**Figure S9**: positive and negative predictive values estimated from the simulation study with five experimental arms as the proportion of treatments which are truly effective changes. Type I error rate/FDR/FWER controlled at 0.1 (one-sided) by the various approaches.

1. Positive predictive value


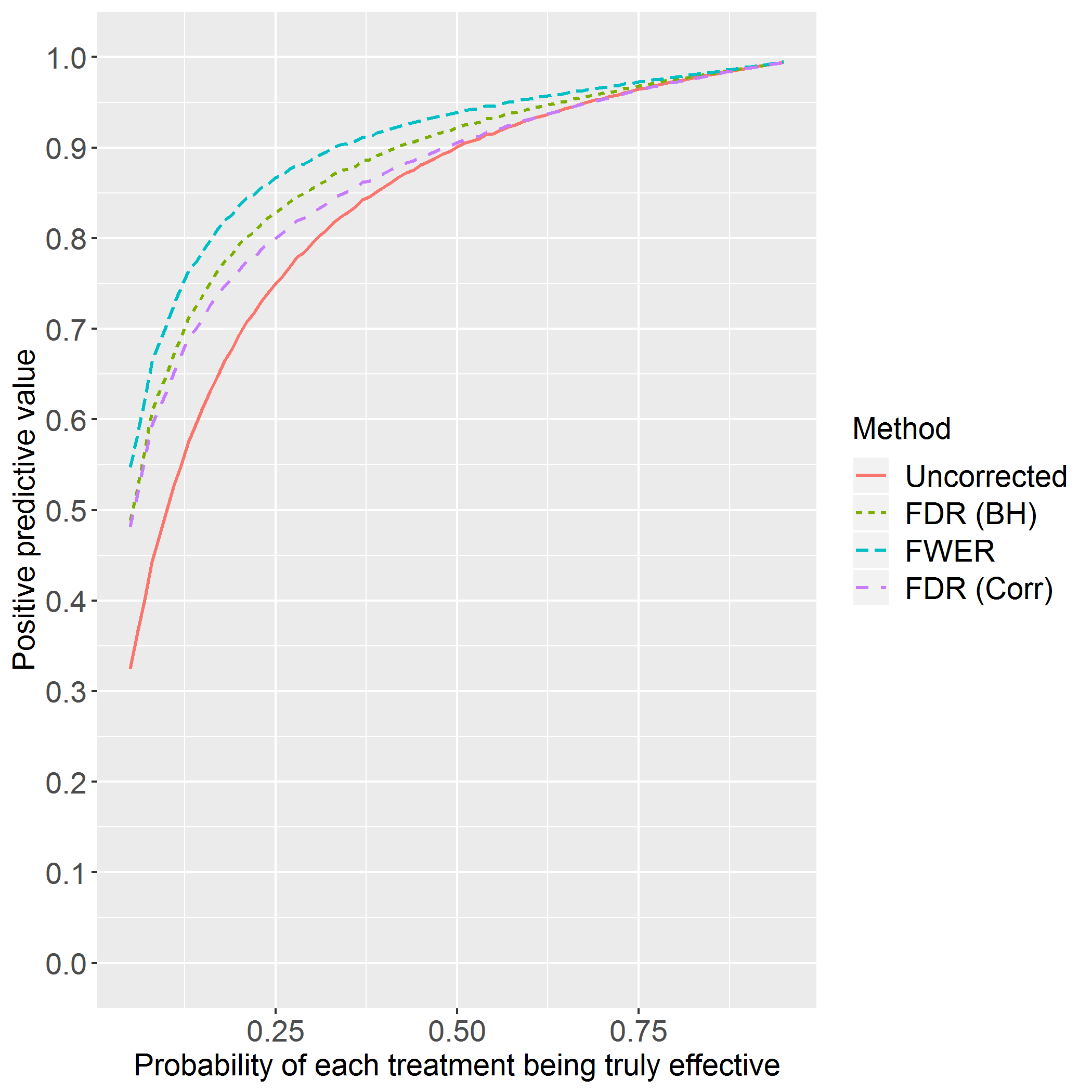


1. Negative predictive value


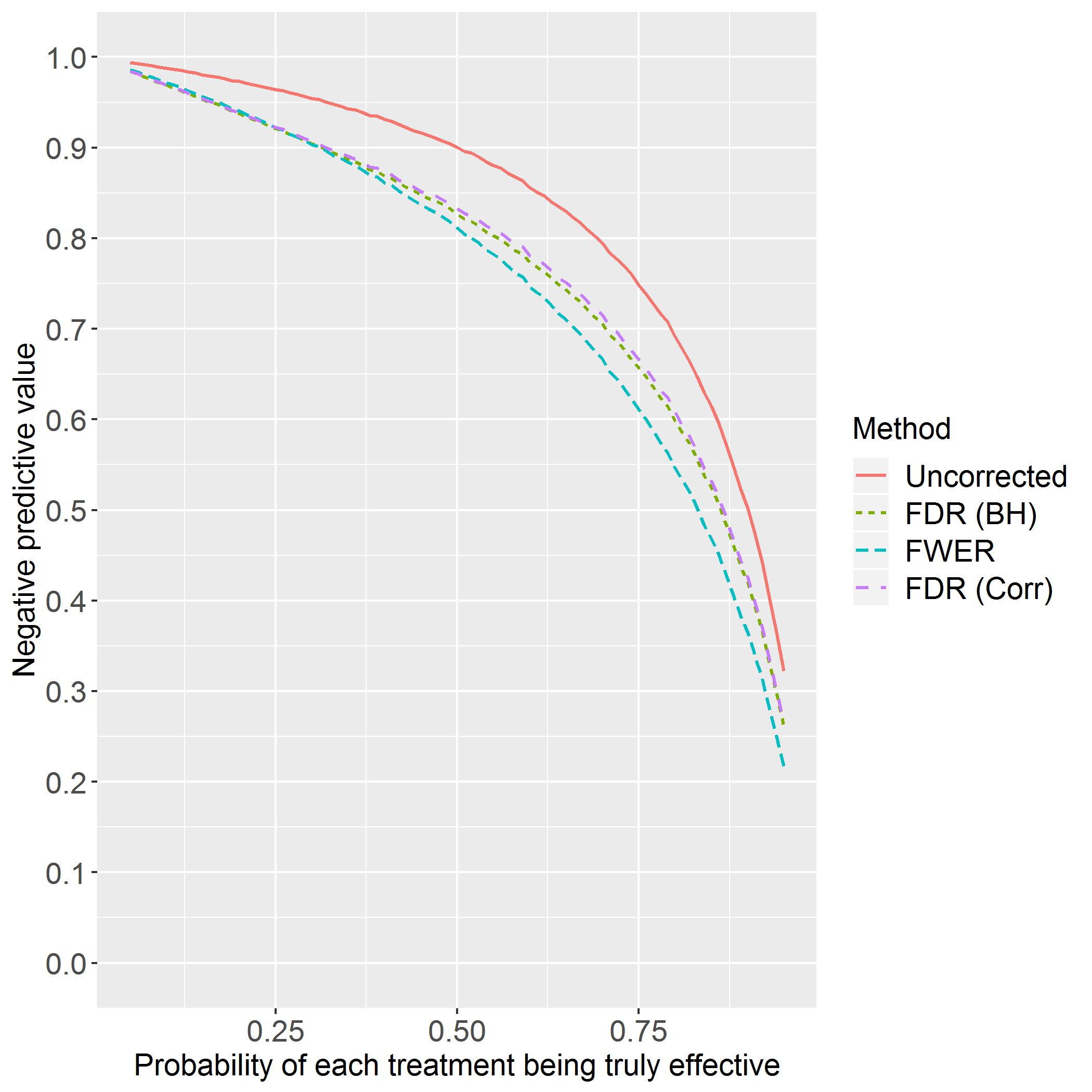


**Figure S10**: positive and negative predictive values estimated from the simulation study with ten experimental arms as the proportion of treatments which are truly effective changes. Type I error rate/FDR/FWER controlled at 0.1 (one-sided) by the various approaches.

1. Positive predictive value


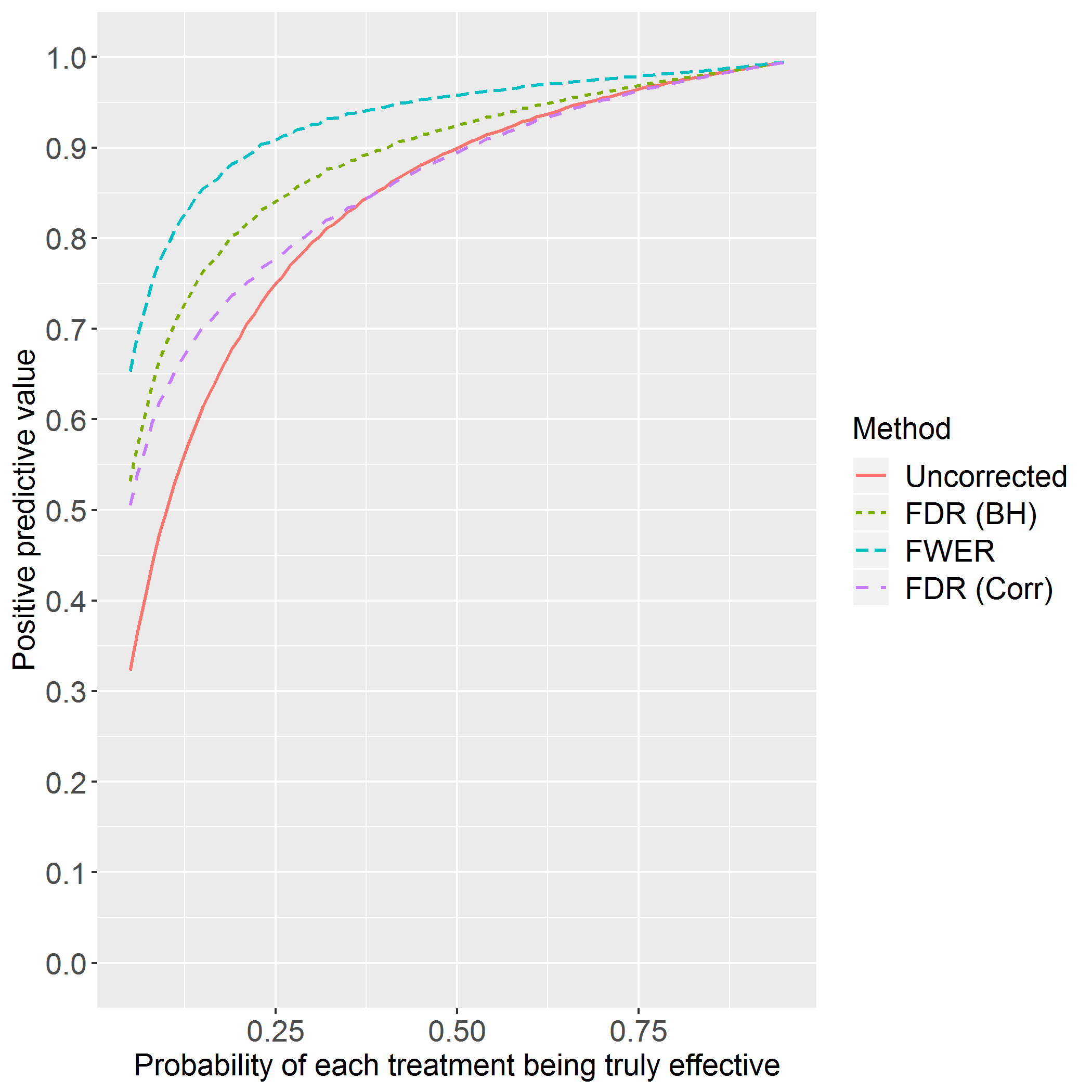


1. Negative predictive value


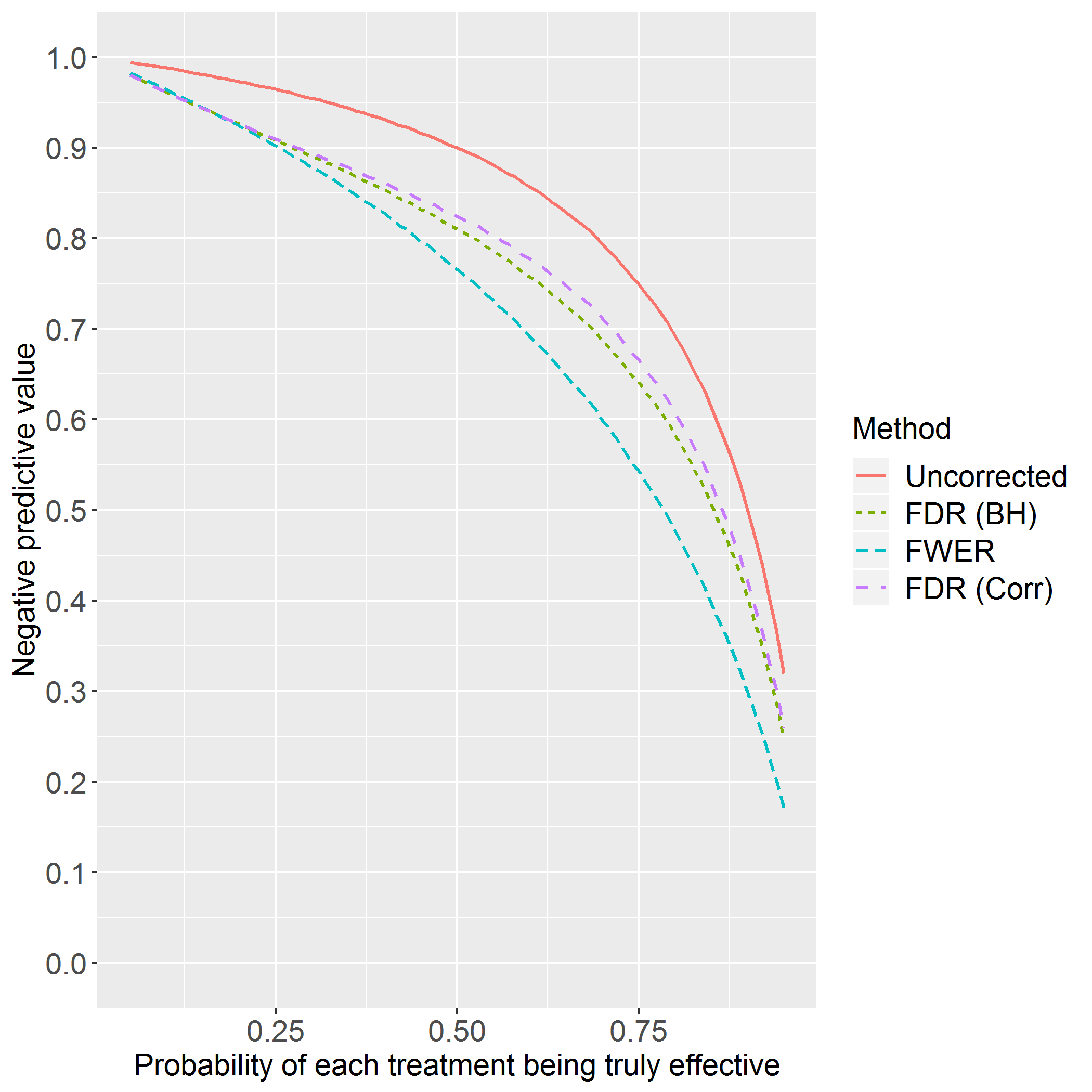


1. Fixed vs random number of effective arms

All the previous results have assumed the number of arms that are effective is a random variable within each trial. Below we show tables comparing the properties when we assume a fixed number of effective arms within each replicate. This is done for K=5, assuming 0, 1, 2, 3, 4 and 5 arms are effective within each replicate. As a comparison we provide properties for the random case with p=0,0.2,0.4,0.6,0.8,1. The properties of the correlated FDR method (proposed by Somerville) are presented in the table under ‘FDR control’. The PPV and NPV are averaged over all replicates, excluding replicates where respectively none and all hypotheses are rejected.

Table S1 – properties of different correction methods when a fixed number of effective arms is assumed.

| Number of effective arms | PPV | | | NPV | | | FWER | | |
| --- | --- | --- | --- | --- | --- | --- | --- | --- | --- |
|  | **Uncorrected** | **FWER control** | **FDR control (Corr)** | **Uncorrected** | **FWER control** | **FDR control (Corr)** | **Uncorrected** | **FWER control** | **FDR control (Corr)** |
| 0 | NA | NA | NA | 1.000 | 1.000 | 1.000 | 0.092 | 0.025 | 0.021 |
| 1 | 0.953 | 0.982 | 0.969 | 0.980 | 0.953 | 0.949 | 0.078 | 0.025 | 0.041 |
| 2 | 0.976 | 0.990 | 0.974 | 0.953 | 0.901 | 0.915 | 0.063 | 0.025 | 0.062 |
| 3 | 0.988 | 0.993 | 0.987 | 0.914 | 0.840 | 0.891 | 0.046 | 0.025 | 0.046 |
| 4 | 0.995 | 0.995 | 0.995 | 0.848 | 0.766 | 0.837 | 0.025 | 0.025 | 0.025 |
| 5 | 1.000 | 1.000 | 1.000 | NA | NA | NA | 0.000 | 0.000 | 0.000 |

Table S2 – properties of different correction methods when a random number of effective arms is assumed.

| Proportion of effective arms | PPV | | | NPV | | | FWER | | |
| --- | --- | --- | --- | --- | --- | --- | --- | --- | --- |
|  | **Uncorrected** | **FWER control** | **FDR control (Corr)** | **Uncorrected** | **FWER control** | **FDR control (Corr)** | **Uncorrected** | **FWER control** | **FDR control (Corr)** |
| 0 | NA | NA | NA | 1.000 | 1.000 | 1.000 | 0.090 | 0.025 | 0.021 |
| 0.2 | 0.919 | 0.971 | 0.960 | 0.977 | 0.951 | 0.955 | 0.077 | 0.026 | 0.039 |
| 0.4 | 0.966 | 0.987 | 0.976 | 0.944 | 0.896 | 0.916 | 0.063 | 0.025 | 0.047 |
| 0.6 | 0.984 | 0.992 | 0.986 | 0.890 | 0.824 | 0.866 | 0.044 | 0.023 | 0.040 |
| 0.8 | 0.994 | 0.996 | 0.994 | 0.766 | 0.694 | 0.751 | 0.023 | 0.016 | 0.023 |
| 1 | 1.000 | 1.000 | 1.000 | NA | NA | NA | 0.000 | 0.000 | 0.000 |

The properties are broadly consistent across the two approaches. Some differences include: 1) higher PPV for the fixed method when the number of effective arms is low and higher NPV when the number of effective is high; 2) higher FWER for the FDR controlling methods in the fixed case.
